# Two new species of *Xanthagaricus* and some notes on *Heinemannomyces* from Asia

**DOI:** 10.3897/mycokeys.28.21029

**Published:** 2017-12-05

**Authors:** Md. Iqbal Hosen, Zong-ping Song, Genevieve Gates, Samantha C. Karunarathna, M. Salahuddin M. Chowdhury, Tai-Hui Li

**Affiliations:** 1 State Key Laboratory of Applied Microbiology Southern China, Guangdong Provincial Key Laboratory of Microbial Culture Collection and Application, Guangdong Institute of Microbiology, Guangzhou 510070, Guangdong, China; 2 South China Botanical Garden, Chinese Academy of Sciences, Guangdong Provincial Key Laboratory of Applied Botany, Guangzhou 510650, Guangdong, China; 3 University of Chinese Academy of Sciences, Beijing 100049, Beijing, China; 4 School of Land and Food, University of Tasmania, Private Bag 54, Hobart, Tasmania 7001, Australia; 5 Key Laboratory for Plant Diversity and Biogeography of East Asia, Kunming Institute of Botany, Chinese Academy of Sciences, Kunming 650201, Yunnan, China; 6 Department of Plant Pathology, Faculty of Agriculture, Sher-e-Bangla Agricultural University, Sher-e-Bangla Nagar, Dhaka-1207, Bangladesh

**Keywords:** *Hymenagaricus*, molecular phylogeny, monophyly, South Asia, taxonomy

## Abstract

*Xanthagaricus flavosquamosus* and *X. necopinatus*, two new species of Agaricaceae, are described and illustrated from Asia. Macroscopically, both species are closely related to each other, but there are obvious micromorphological and molecular differences between them. Morphological and phylogenetic data showed that the two new species are distinct from other known species of the genus *Xanthagaricus*. *Xanthagaricus flavosquamosus* from China is characterized by its small, yellow basidiomata, short clavate cheilocystidia, epithelial pileipellis, and verrucose basidiospores measuring 5–5.5 × 3–3.5 μm. *Xanthagaricus necopinatus* from Bangladesh is characterized by having small, yellow basidiomata, a fugacious annulus, clavate to narrowly clavate cheilocystidia, epithelial pileipellis, and rugulose-rough basidiospores measuring 4–5 × 2.7–3.2 μm. In addition to the new species, a *Heinemannomyces* collection from China is reported. Morphological data and molecular phylogenetic analyses fully support the Chinese collection being *Heinemannomyces splendidissimus*, a species of Agaricaceae, originally described from Southeast Asia. Detailed descriptions, color photos and illustrations of the three species are presented. A key to the genus *Xanthagaricus* occurring in Bangladesh and China is provided.

## Introduction

*Xanthagaricus* (Heinem.) Little Flower, Hosag. & T.K. Abraham is mainly characterized by small basidiomata with squamulose pileus, epithelial pileipellis, and more or less yellow-colored basidiospores (Little Flower et al. 1997; Hosen et al. 2017). This genus was originally placed in Hymenagaricus Heinem. subgenus Xanthagaricus Heinem. by Heinemann and Little Flower (1984) with the type species *X. flavidorufus* (Berk. & Broome) Heinem. & Little Flower. Heinemann (1981) erected *Hymenagaricus* as an independent genus in the family Agaricaceae with the type species *H. hymenopileus* (Heinem.) Heinem., and is characterized by a squamulose pileus, a hymeniform pileipellis, and brown basidiospores (Heinemann and Little Flower 1984, Ge et al. 2008, Hosen et a. 2017). The epithelial pileipellis and yellow basidiospores of the subgenus Xanthagaricus did not fit completely with the genus circumscription of *Hymenagaricus*. Subsequently, the subgenus Xanthagaricus was elevated to the genus level in Agaricaceae (Little Flower et al. 1997).

Species in the genus *Xanthagaricus* are saprotrophic, and mainly distributed in Asia and South Africa. For instance, 11 species from India (Heinemann and Little Flower 1984, Little Flower et al. 1997), four species from Sri Lanka (Heinemann and Little Flower 1984, Pegler 1986, Little Flower et al. 1997), two species from Africa (Reid and Eicker 1995, 1998), one species each from mainland China (Hosen et al. 2017), Pakistan (Hussain et al. 2017), and Taiwan, China (Ge et al. 2008) have so far been validly reported. However, Asia has more than the currently known species of *Xanthagaricus*, as sequences of several species of this genus are available in GenBank, especially from Thailand and Malaysia. Index Fungorum (http://www.indexfungorum.org/Names/Names.asp) lists 12 taxa of *Xanthagaricus*. However, a recent study by Hussain et al. (2017) has transferred six species to *Xanthagaricus* from *Hymenagaricus*.

Watling (1998) circumscribed *Heinemannomyces* as an independent monotypic genus in the family Agaricaceae from specimens collected in Malaysia and Thailand. Since then, no additional species with detailed descriptions and geographical extensions of the genus *Heinemannomyces* have been reported so far. *Heinemannomyces* is distinguished by its medium-sized basidiomata, extremely woolly-arachnoid veils on the pileus surface composed of cylindrical cells, leaden gray to dark blue lamellae, and reddening context when injured (Watling 1998). Phylogenetically, *Heinemannomyces* is closely related to *Hymenagaricus*, but can be differentiated by its morphology.

In this study, three collections of *Xanthagaricus* and *Heinemannomyces* from China, and one collection of *Xanthagaricus* with several basidiomata from tropical Bangladesh were examined. Based on macromorphology, both East Asian and South Asian *Xanthagaricus* collections could be the same species. However, careful microscopic observations along with molecular data revealed that they are not conspecific, but represent undescribed species within *Xanthagaricus*. In addition, a brief description from the Chinese collection of *Heinemannomyces* is provided along with molecular data. With the inclusion of the two new species of *Xanthagaricus* in this study and another two recently described new species, namely *X. caeruleus* Iqbal Hosen, T.H. Li & Z.P. Song (Hosen et al. 2017) and *X. pakistanicus* Hussain, Afshan & Ahmed (Hussain et al. 2017), the number of known species of this genus increases to 22.

## Materials and methods

### Morphological studies

Specimens of *Xanthagaricus* and *Heinemannomyces* were collected from south China and Bangladesh (*Xanthagaricus*). The examined specimens were deposited in the Fungal Herbarium of the Guangdong Institute of Microbiology (GDGM), Guangzhou, China, and in the private herbarium (PHI) of the first author. Macromorphological descriptions were based on the field notes and photographs. Color codes and names follow Kornerup and Wanscher (1978).

Micromorphological observations were made from the dried specimens. Line drawings were freehand. Water, 5% aqueous KOH (w/v), and Congo Red were used as mounting media; Melzer’s solution was used to check any amyloid reaction of basidiospores and tissues. In the descriptions of basidiospore measurements, the notation [n/m/p] was used, which means *n* basidiospores from *m* basidiomata of *p* collections. Dimensions for basidiospores are given as (a–)b–c(–d), in which ‘b–c’ contains a minimum of 90% of the measured values and extreme values ‘a’ and ‘d’ are given in parentheses, whenever necessary. Q denotes the length/width ratio of a measured basidiospore, Q_m_ denotes the average of *n* basidiospores and SD is their standard deviation. Results are presented as Q_m_ ± SD. Basidiospores were also observed using a scanning electron microscope (SEM) following the protocol of Hosen et al. (2013).

### Molecular studies

Protocols for genomic DNA extraction, PCR amplification, and sequencing followed Hosen et al. (2013). ITS1-F/ITS4 (White et al. 1990) and LROR/LR5 (Vilgalys and Hester 1990) primer pairs were used for the amplification of the nuclear ribosomal internal transcribed spacer (ITS) region and partial sequence of nuclear ribosomal large subunit (28S) domains D1 and D2.

A total of 52 sequences (36 for ITS and 16 for 28S, Table 1) of Agaricaceae was retrieved from GenBank based on NCBI blast search results and recent publications (Ge et al. 2008, Vellinga et al. 2011, Ge and Yang 2017, Hosen et al. 2017, Hussain et al. 2017), and then combined with the newly generated ITS and 28S sequences of *Clarkeinda trachodes* (Berk.) Singer, *Xanthagaricus* and *Heinemannomyces*. Each individual dataset, either ITS or 28S, was aligned in MAFFT v.6.8 (Katoh et al. 2005) separately with default settings, and manually edited in BioEdit v.7.0.9 (Hall 1999). ITS and 28S datasets was then concatenated using Phyutility (Smith and Dunn 2008) for further phylogenetic analyses, and treated here as a ITS-28S dataset. The combined dataset (ITS-28S) was used for the recognition of the new species in *Xanthagaricus* and to find out their relationships with allied genera in Agaricaceae. Maximum Likelihood (ML) was performed using RAxML v.7.2.6 (Stamatakis 2006). As RAxML only supports the GTR model of nucleotide substitution, the GTRGAMMAI model was used for phylogenetic analyses, and statistical support values were obtained using nonparametric bootstrapping (BS) with 1000 replicates. *Chlorophyllum rachodes* (Vittad.) Vellinga was chosen as the outgroup.

**Table 1.** List of fungal taxa of Agaricaceae and their GenBank accession numbers used in molecular phylogeny.

Name of the species	Voucher/collection no.	Country	GenBank accession no.	
			ITS	28S
Agaricus aff. campestris	Murphy 6242	USA	HM488744	–
*Agaricus bisporatus*	Contu1	–	AF432882	–
*Agaricus bohusii*	LAPAG562	–	KR006613	KR006613
*Agaricus deserticola*	S. Smith	USA	HM488747	–
*Agaricus diminutivus*	Vellinga 2360	USA	AF482831	AF482877
*Agaricus megacystidiatus*	MFLU 12–0137	Thailand	NR_119953	–
*Agaricus rotalis*	ecv3768	USA	HM488746	HM488792
Agaricus sp.	BAB–5059	India	KR155104	
*Agaricus* sp.	CA833	Thailand	JF727858	–
*Agaricus* sp.	C3182	Togo	KJ540956	–
*Agaricus* sp.	NTS113	Thailand	JF514531	–
*Chlorophyllum rachodes*	Vellinga 2106	Netherlands	AF482849	–
*Clarkeinda trachodes*	ecv3838	Thailand	M488750	HM488771
***Clarkeinda trachodes***	**Iqbal 806**	**Bangladesh**	–	**MG462712**
*Coniolepiota spongodes*	ecv3898	Thailand	HM48875	–
*Coniolepiota spongodes*	HKAS 77574	Bangladesh	KC625531	KC625530
*Eriocybe chionea*	ecv3560	Thailand	HM488752	HM488773
*Heinemannomyces splendidissimus*	ecv3586	Thailand	HM488760	HM488769
*Heinemannomyces splendidissimus*	zrl3043	Thailand	JF691559	–
***Heinemannomyces splendidissimus***	**GDGM 46633**	**China**	**MF621038**	**MF621039**
***Heinemannomyces splendidissimus***	**GDGM 46633**	**China**	–	**MF621040**
*Hymenagaricus ardosiicolor*	LAPAF9	Togo	JF727840	–
*Hymenagaricus ardosiicolor*	isolateZ4	Tanzania	KM360160	–
Hymenagaricus cf. kivuensis	BR6089	Burundi	KM982454	
*Hymenagaricus* sp.	CA833	Thailand	JF727858	–
*Hymenagaricus* sp.	zrl3103	Thailand	KM982450	KM982452
*Hymenagaricus* sp.	CA801	Thailand	JF727859	–
*Hymenagaricus* sp.	LD2012186	Thailand	KM982451	KM982453
*Pseudolepiota zangmui*	Ge2106^*^	China	KY768927	–
*Pseudolepiota zangmui*	MFLU100515	Thailand	KX904355	–
*Xanthagaricus caeruleus*	GDGM 50651^*^	China	MF039088	MF039086
*Xanthagaricus caeruleus*	GDGM 50794	China	MF039089	MF039087
*Xanthagaricus epipastus*	zrl 3045	Thailand	HM436649	HM436609
***Xanthagaricus flavosquamosus***	**GDGM 50913**	**China**	**MF351627**	–
***Xanthagaricus flavosquamosus***	GDGM 50918^*^	**China**	**MF351629**	**MF351631**
***Xanthagaricus flavosquamosus***	**GDGM 50924**	**China**	**MF351628**	–
***Xanthagaricus necopinatus***	Iqbal–821 (GDGM 46632^*^, PHI–12^#^)	**Bangladesh**	**MF351626**	**MF351630**
*Xanthagaricus pakistanicus*	LAH SH 207	Pakistan	KY621555	–
*Xanthagaricus pakistanicus*	HUP SH 315	Pakistan	KY621556	–
*Xanthagaricus* sp.	TL6025	Malaysia	AF482835	AF482879
*Xanthagaricus* sp.	ecv3807	Thailand	HM488761	HM488770
*Xanthagaricus taiwanensis*	HKAS 42545	Taiwan, China	DQ490633	DQ089016
*Xanthagaricus taiwanensis*	C.M. Chen 3636^*^	Taiwan, China	DQ006271	DQ006270

Highlighted in bold are newly generated sequences in this study.

^*^holotype

^#^isotype

## Results

### Molecular phylogenetic results

**Figure 1.**
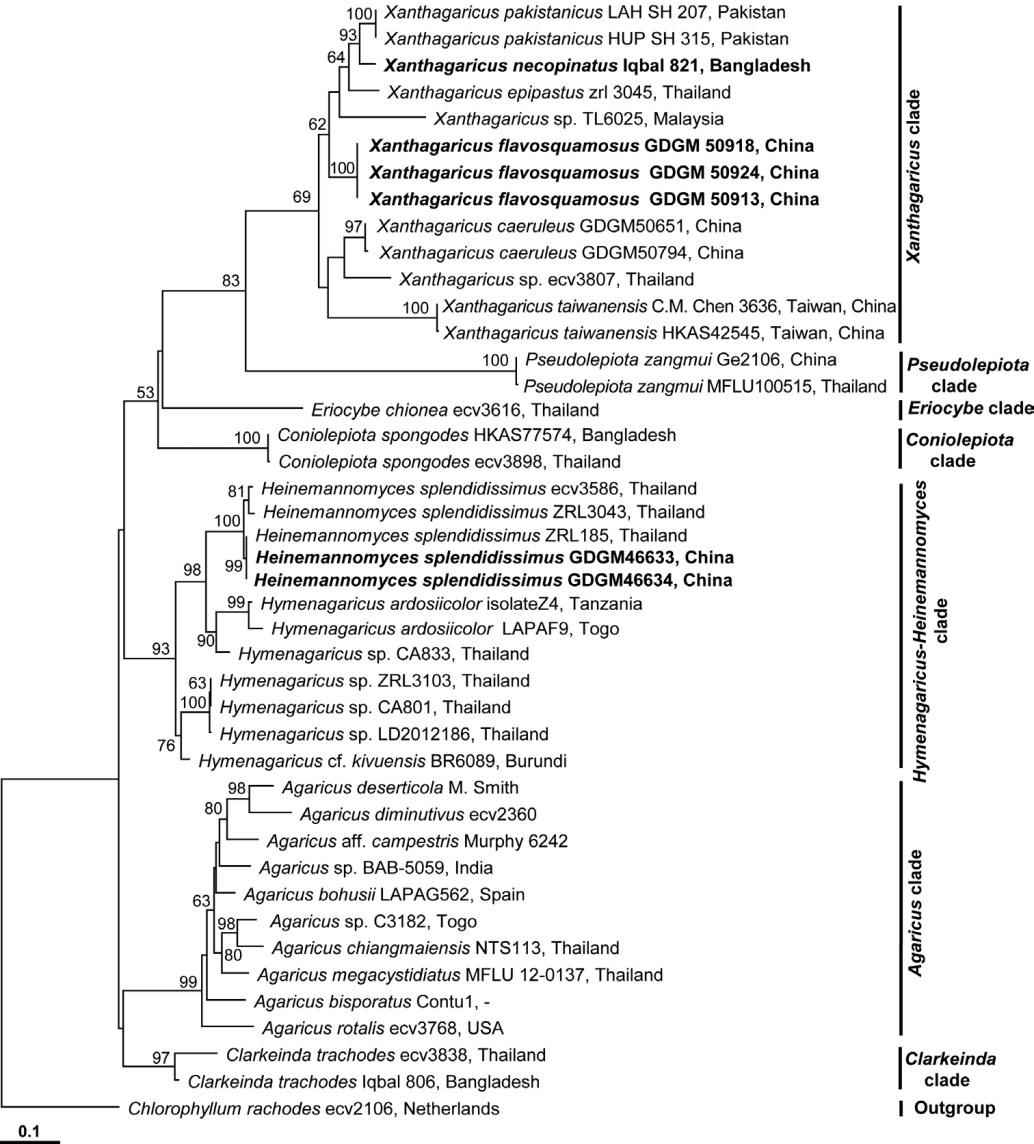
Phylogenetic relationships of *Xanthagaricus* species and its allied genera inferred from ITS-28S data using ML method. RAxML bootstrap support values (ML BS, ≥50%) are indicated on the branches at nodes. The two new species of *Xanthagaricus* from Bangladesh and China, and *Heinemannomyces splendidissimus* from China, are highlighted in bold on the tree. Herbarium or voucher specimen numbers and country names are provided after the species name. *Chlorophyllum rachodes* is rooted as the outgroup. Bar: indicates 0.1 expected change per site per branch.

A total of 10 nuclear ribosomal RNA gene sequences (five each for ITS and 28S) was generated from the newly collected materials of *C. trachodes*, *Heinemannomyces* and *Xanthagaricus*, and deposited in GenBank (Table 1). In the aligned ITS-28S dataset, sequences of the 43 samples were included with 1663 nucleotide sites (784 for ITS and 879 for 28S, gaps included) for each sample, of which 1186 were constant characters, 384 were parsimony informative characters, and 93 were parsimony uninformative characters. The resulting aligned dataset has been deposited in TreeBASE (http://purl.org/phylo/treebase/phylows/study/TB2:S21521). In the combined ITS-28S ML tree (Fig. 1), the proposed two new species are distinct, the collection from Bangladesh is a close relative to *X. pakistanicus* with strong BS support value (97% ML BS), while the Chinese collection is not sister to any single species. Both clustered together with *X. epipastus* (Berk. & Broome) Hussain, *X. taiwanensis* (Zhu L. Yang, Z.W. Ge & C.M. Chen) Hussain, *X. caeruleus*, and two unnamed species of the same genus. The result of the phylogenetic analysis is presented in Fig. 1.

## Taxonomy

### Xanthagaricus flavosquamosus

Taxon classificationFungiAgaricalesAgaricaceae

T.H. Li, Iqbal Hosen & Z.P. Song sp. nov.

822730

Figs 2a–b , 3 , 5b

#### Diagnosis.

Closely related to *X. epipastus* and *X. subepipastus* but differs in having larger basidiospores with verrucose surface, short but broadly clavate cheilocystidia, and found on the ground covered by fallen needles or debris of *Pinus* sp.

#### Typification.

CHINA, Jiangxi Province, Jiulong Provincial Forest Park, 25 August 2015, Ming Zhang, Jun Ping Zhou & Hao Huang (GDGM 50918, holotype).

#### Etymology.

The species epithet “*flavosquamosus*” (Lat.) refers to the yellow squamules on the pileus surface.

#### Description.

*Basidiomata* small-sized. *Pileus* 8–13 mm broad, at first hemispherical to convex, then plano-convex to nearly applanate with age, yellow (2A4–7) to vivid yellow (2A8), lemon yellow or mustard yellow (3B8, 3B6), more or less yellow-brown to grayish brown at centre, concentrically fibrillose-squamulose, sometimes woolly to matted squamulose on the surface, more densely and darker at centre; margin incurved with appendiculate, often lacerated velar remnants, concolorous with the squamules; context 0.8 mm thick at the pileus center, elsewhere thin, no color change when cut or injured. *Lamellae* free, depressed around the stipe, broadly ventricose, yellowish white (3A2) to light pinkish white (10A2), with crenulate margin; 3–4 tiers of lamellulae. *Stipe* 20–30 × 1.5–2 mm, equal, central, cylindrical, slightly curved, fistulose, pale yellow (3A3) to slightly grayish yellow (3B3), some scattered squamules or scales on surface, with white mycelial tufts at base. *Annulus* absent. *Odor and taste* unknown.

*Basidiospores* [60/3/3] 5–5.5(–6) × 3–3.5 µm, [mean length = 5.38 µm, mean width = 3.25 µm, Q = (1.51–)1.61–1.68(–1.71), Q_m_ = 1.65 ± 0.052], ellipsoid to broadly ellipsoid, slightly thick-walled (0.5 µm), smooth under light microscope but minutely verrucose or warty under SEM, pale yellow to yellowish brown in H_2_O and 5% KOH, inamyloid. *Basidia* 10–12 × 5–6 µm, clavate, pale yellow in H_2_O, hyaline, thin-walled, 4-spored, with sterigmata up to 3 µm long. *Lamellar trama* regular to subregular, composed of thin-walled cylindrical hyphae 4–8 µm wide. *Cheilocystidia* 7–15 × 6–9 µm, abundant, clavate to broadly clavate, sometimes slightly fusoid to obovate, smooth, hyaline, thin-walled. *Pleurocystidia* absent. *Pileipellis* (squamules on pileus) epithelial, composed of agglutinated globose to subglobose, rarely clavate to ellipsoidal thin-walled cells, terminal cells 6–12 × 6–10 µm, slightly encrusted, with some vacuolar pigments when observed in KOH or H_2_O. *Caulocystidia* not found. *Stipe trama* composed of parallel hyphae 3–8 µm wide, yellowish brown in mass but pale yellow or subhyaline individually. *Clamp connections* absent in all tissues.

#### Habit, habitat and distribution.

Gregarious to scattered, ground covered with fallen needles or debris of *Pinus* sp., currently only known from Jiangxi Province of China.

#### Additional specimens examined.

CHINA, Jiangxi Province, Jiulong Provincial Forest Park, 26 Aug 2015, Ming Zhang, Jun Ping Zhou & Hao Huang (GDGM 50924); same location, 26 Aug 2015, Ming Zhang, Jun Ping Zhou & Hao Huang (GDGM 50613b).

**Figure 2.**
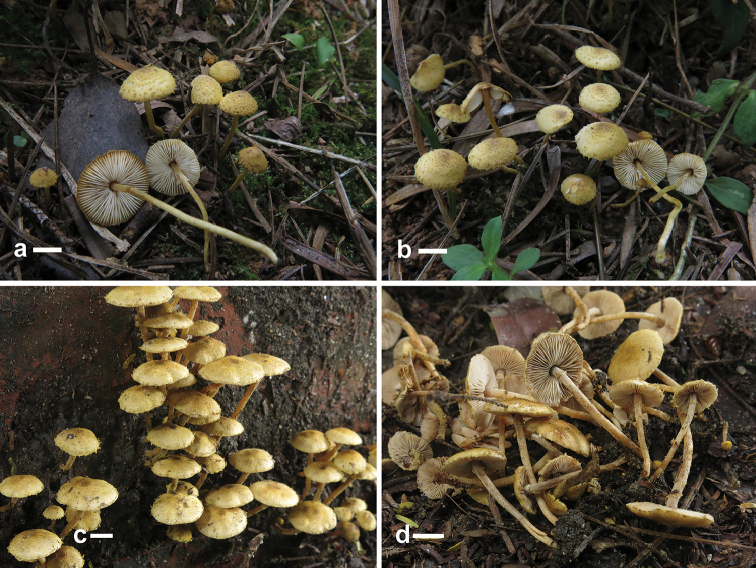
Basidiomata of *Xanthagaricus* species. **a, b** Basidiomata of *X. flavosquamosus* (**a, b** GDGM 50918, holotype) **c, d** Basidiomata of *X. necopinatus* (GDGM 46632, holotype; PHI-12, isotype). Scale bars: 5 mm.

### Xanthagaricus necopinatus

Taxon classificationFungiAgaricalesAgaricaceae

Iqbal Hosen, T.H. Li, & G.M. Gates sp. nov.

822731

Figs 2c, d , 4 , 5b

#### Diagnosis.

Morphologically similar to *X. flavosquamosus* but differs in the presence of a fugacious annulus, smaller and denser squamules, comparatively smaller basidiospores with rugulose-rough surface, clavate to narrowly clavate cheilocystidia.

#### Typification.

BANGLADESH, Dhaka Division, Sher-e-Bangla Nagar, Chondrima Uddan, 21 Aug 2014, Iqbal 821 (GDGM 46632, holotype; PHI-12, isotype).

#### Etymology.

The species epithet “*necopinatus*” (Lat.) means unexpected, refers to the unexpected, surprising habitat of the collection, which was found on a concrete wall.

**Figure 3.**
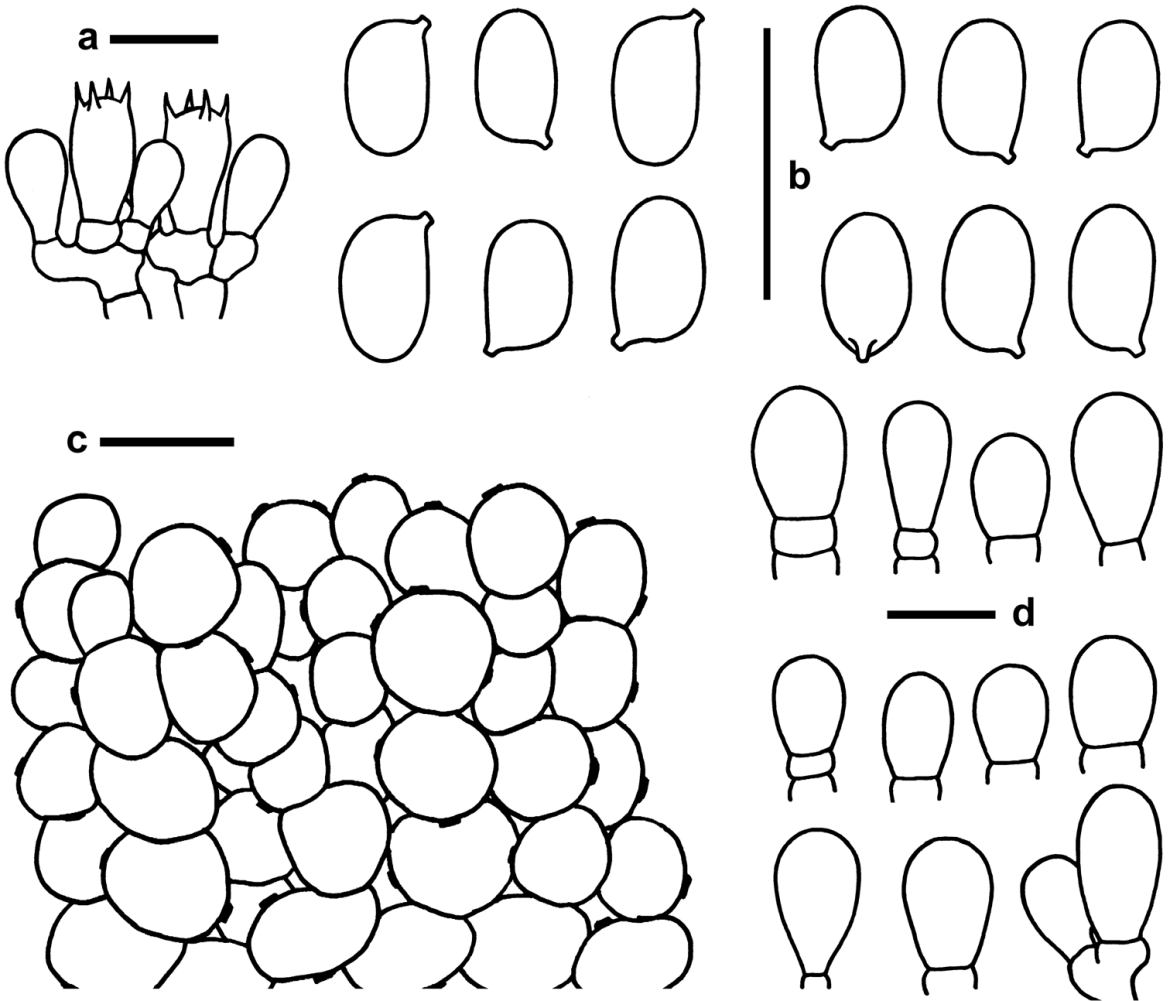
Microscopic features of *Xanthagaricus flavosquamosus* (GDGM 50918, holotype). **a** Basidia with basidioles **b** Basidiospores **c** Epithelial pileipellis with encrusted wall. **d** Cheilocystidia. Scale bars: 10 µm.

#### Description.

*Basidiomata* small-sized. *Pileus* 10–15 mm broad, hemispherical, convex to plano-convex, yellow (2A4–7) to vivid yellow (2A8), maize yellow (4A6), light olive yellow (3D3–4) to pale brown (5D4) at disc, with yellow (3A4) to yellowish brown (5D8, 5E8) squamulose or finely fibrillose squamules on the surface, more concentrated and darker at center but scattered elsewhere; margin incurved with appendiculate velar remnants, concolorous with the pileus squamules; context 0.7 mm thick at pileus center, elsewhere thin, unchanged when cut or injured. *Lamellae* free, depressed around the stipe, yellowish white (3A2) to pinkish white (10A2), light brownish gray (6C3, 6D3), with crenulate edge, broadly ventricose; lamellulae commonly with 3–4 tiers. *Stipe* 18–28 × 1.5–2 mm, equal to slightly attenuated towards base, central, cylindrical, slightly curved, fistulose, yellowish brown (5D4) to dull brown (5C2), with some scattered squamules on surface; squamules more concentrated toward apex. *Annulus* very thin and tiny, superior, fugacious, often gone due to handling or with age. *Odor and taste* unknown.

*Basidiospores* [60/3/1] 4–5 × 2.7–3.2 µm, [mean length = 4.45 µm, mean width = 2.98 µm, Q = (1.31–)1.41–1.64(–1.72), Q_m_ = 1.49 ± 0.064], ellipsoid to ovoid-ellipsoid, slightly thick-walled (0.5 µm), inamyloid, smooth under light microscope but rugulose-rough surface under SEM, yellow to yellowish brown in H_2_O and 5% KOH. *Basidia* 13–17 × 5–6 µm, clavate to narrowly clavate, pale yellow in H_2_O, thin-walled, 4-spored, with sterigmata up to 2 µm long. *Lamellar trama* regular to subregular, composed of thin-walled cylindrical hyphae, 4–8 µm wide. *Cheilocystidia* 15–20 × 4–6 µm, abundant, clavate to narrowly clavate, sometimes narrowly fusoid, smooth, hyaline, thin-walled. *Pleurocystidia* absent. *Pileipellis* (squamules on pileus) epithelial, composed of agglutinated globose, subglobose to broadly ellipsoid, rarely clavate cells, terminal cells measuring 9–15 × 6–10 µm, slightly encrusted, with some vacuolar pigments when observed in KOH or H_2_O. *Caulocystidia* sometimes present, cylindro-clavate to narrowly clavate measuring 18–25 × 5–7 µm, thin-walled, smooth, hyaline. *Stipe trama* composed of parallel hyphae 4–10 µm wide, yellowish brown in mass but pale yellow to subhyaline individually. *Clamp connections* absent in all tissues.

#### Habit, habitat and distribution.

Scattered, clustered on a concrete wall, currently only known from Bangladesh.

**Figure 4.**
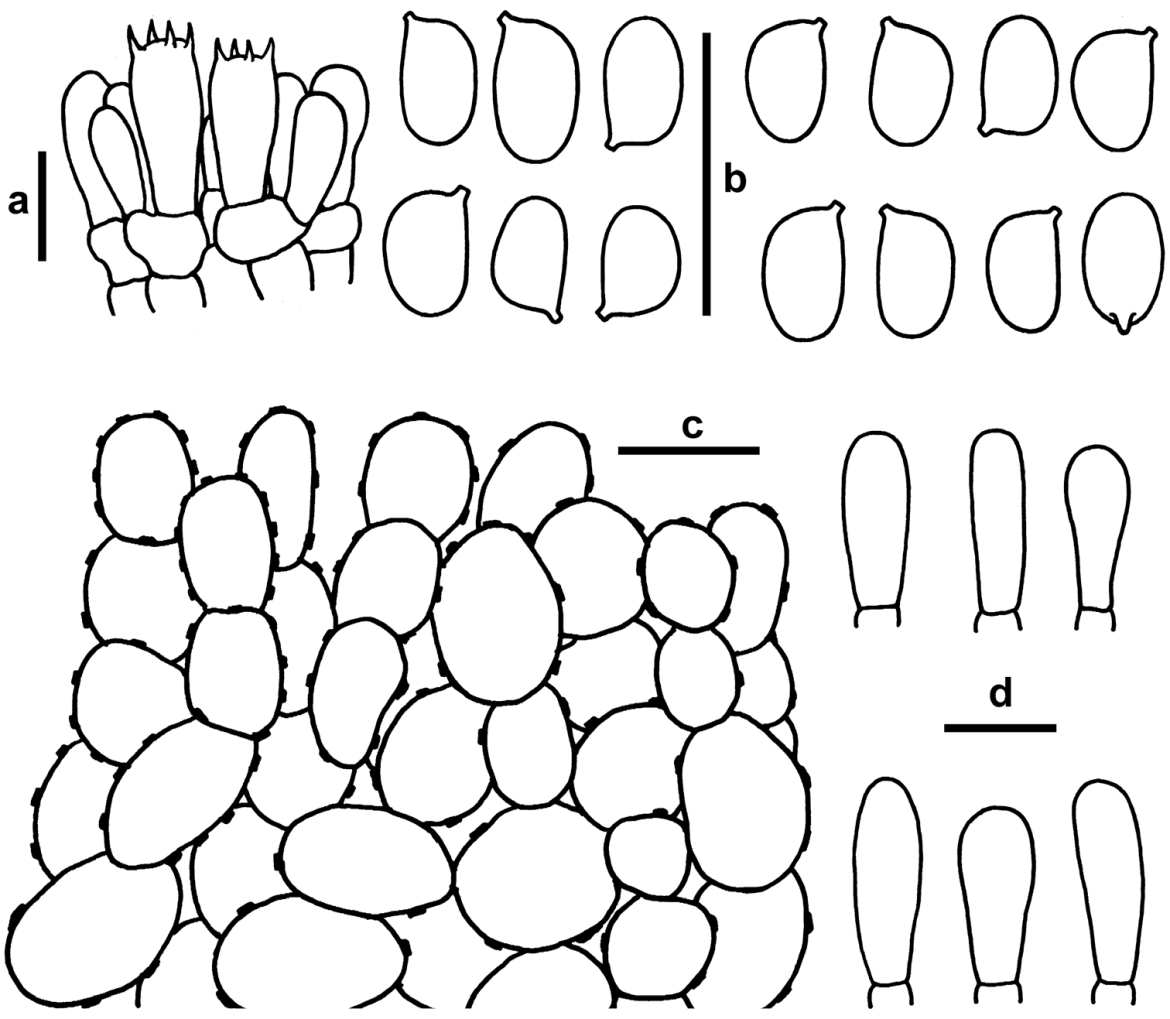
Microscopic features of *Xanthagaricus necopinatus* (GDGM 46632, holotype; PHI-12, isotype). **a** Basidia with basidioles **b** Basidiospores **c** Epithelial pileipellis with encrusted wall **d** Cheilocystidia. Scale bars: 10 µm.

**Figure 5.**
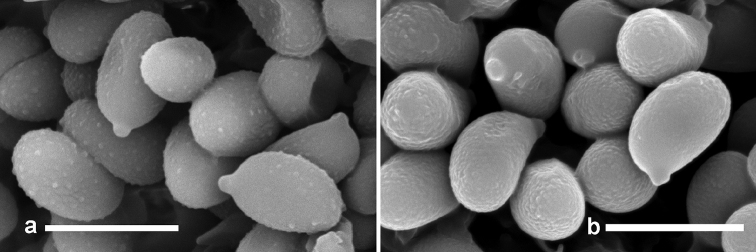
SEM of basidiospores of *Xanthagaricus* spp. **a** SEM basidiospores of *X. flavosquamosus* (GDGM 50918, holotype) **b** SEM of basidiospores of *X. necopinatus* (PHI-12, isotype). Scale bars: 5 µm.

### Heinemannomyces splendidissimus

Taxon classificationFungiAgaricalesAgaricaceae

Watling, Belg. J. Bot. 131(2): 135 (1998)

Figs 6 , 7

#### Description.

*Basidiomata* medium-sized to large. *Pileus* 35–65 mm broad, at first hemispherical, then convex to applanate with age, sometimes depressed at disc, pileus surface covered by snuff brown, chestnut brown, purple-brown or grayish red (9B4, 9C4, 10CD4), woolly-floccose or woolly arachnoid velar remnants, usually darker at center, outer zone showing dull white to whitish background when the velar remnants vanish; margin incurved with slightly appendiculate velar remnants, often slightly lacerated; context 3–4 mm thick at pileus centre, elsewhere thin, changes from white to slightly reddening when cut or injured. *Lamellae* free, depressed around the stipe, broadly ventricose, bluish gray to leaden gray (19B3, 18C3) when young, becoming dark blue (19E4–7) to bluish gray (19D3–5) when mature; 3–4 tiers of lamellulae. *Stipe* 50–60 × 5–6 mm, central, cylindrical, slightly tapering towards the base, floccose-squamulose all over the stipe, often vanish from handling or rain, with lighter shade of the pileus color, fistulose; *stipe context* slightly reddening when cut or injured. *Annulus* delicate, fugacious. *Odor and taste* unknown.

*Basidiospores* [60/3/3] (5.5–)6–6.5(–7) × 3.5–4.5(–5) µm, [mean length = 6.25 µm, mean width = 4.15 µm, Q = 1.42–1.55(–1.63), Q_m_ = 1.50 ± 0.043], ellipsoid to ovoid-ellipsoid, inamyloid, slightly thick-walled (0.5 µm), smooth, dark brown, grayish brown to slightly leaden gray in H_2_O and 5% KOH. *Basidia* 13–19 × 7–9 µm, clavate to broadly clavate, colorless or pale yellow in H_2_O and KOH, 4-spored, rarely 2-spored, with sterigmata up to 2 µm long. *Lamellar trama* regular to subregular, composed of thin-walled cylindrical hyphae 4–8 µm wide. *Cheilocystidia* 15–22 × 6–10 µm, abundant and scattered colorless, clavate to cylindro-clavate, sometimes narrowly fusoid, smooth, hyaline, thin-walled. *Pleurocystidia* absent. *Pileus* hyaline or pale yellow, 4–10 wide hyphae; refractive hyphae very common, 5–8 µm wide. *Pileipellis* (woolly-floccose squamules on pileus) a complex of hyphal types, interwoven, loosely arranged, brick red in mass but hyaline to light red or pale red individually when observed in H_2_O and KOH, sometimes slightly puffy or swollen in some portion of some hyphae, smooth, thin-walled, cylindrical hyphae 4–10 µm wide; terminal elements measuring 20–50 × 4–10 µm. *Stipitipellis* similar to pileipellis but with paler color and slightly narrower hyphae measuring 3–8 µm wide. *Stipe trama* composed of parallel hyphae 4–9 µm wide, hyaline; refractive hyphae sometimes present 3–5 µm wide. *Clamp connections* not found in any tissue.

#### Habit, habitat and distribution.

Solitary, scattered on the ground; known from Malaysia, Thailand, and now China.

#### Specimens examined.

CHINA, Guangdong Province, Shantou City, Nanao Island, 8 May 2015, Iqbal, Tai-Hui Li & Ting Li (GDGM 46633); same location, 9 May 2015, Iqbal, Tai-Hui Li & Ting Li (GDGM 46634, GDGM46635).

**Figure 6.**
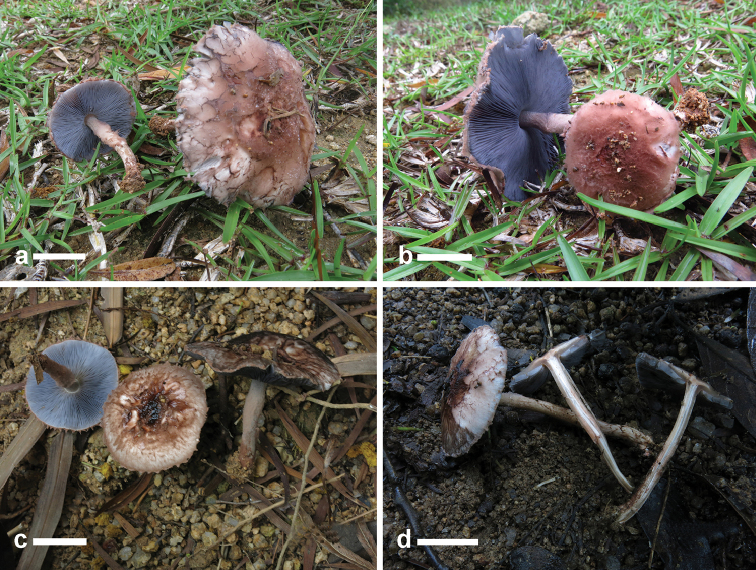
Basidiomata of *Heinemannomyces splendidissimus*. **a** Basidiomata showing leaden-blue lamellae and floccose pileus surface (GDGM 46633) **b** Basidiomata showing dark blue lamellae and floccose pileus surface (GDGM 46633) **c** Basidiomata showing blue lamellae and slightly depressed pileus disc (GDGM 46634) **d** Basidiomata showing pileus surface and a reddening context when cut (GDGM 46635). Scale bars: 20 mm.

**Figure 7.**
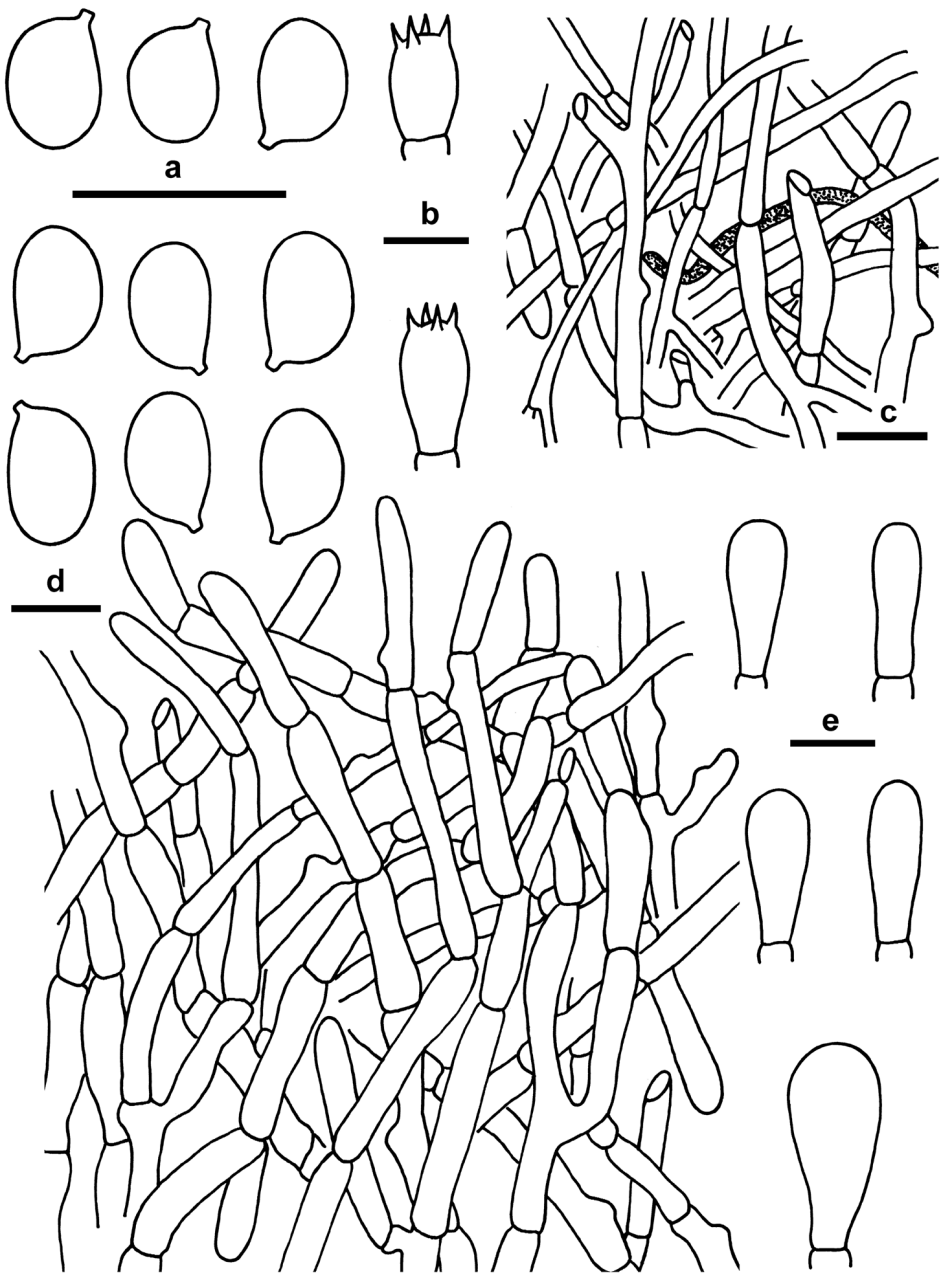
Microscopic features of *Heinemannomyces splendidissimus* (GDGM 46633). **a** Basidiospores **b** Basidia **c** Elements from stipe surface **d** Pileipellis **e** Cheilocystidia. Scale bars: 10 µm.

## Discussion

Little Flower et al. (1997) defined *Xanthagaricus* to include taxa with “basidiomata small, pileus with characteristic woolly squamules and appendiculate margin; lamellae free, brown when mature; stipe cylindrical, slender, fistulose, slightly broader at the apex, veil absent; context thin, sometimes becoming vinaceous red on bruising; spore print brown, spores subglobose to ellipsoid, smooth, thick-walled, brown with yellowish tinge; lamellar edge heteromorphous; cheilocystidia present; hymenophoral trama regular to subregular; pileal surface a disrupted epicutis of radial hyphae with plenty of spherical or subspherical cells at the scales; clamp connections absent”. In spite of that, some species of *Xanthagaricus* do not warrant the generic circumscription on account of lamellae color (ink-blue in *X. caeruleus*), presence of an annulus (tiny and fugacious annulus in *X. necopinatus*), basidiospore color (greenish gray to grayish brown in *X. caeruleus*) with either smooth (*X. caeruleus*) or ornamented basidiospores (*X. flavosquamosus*, *X. necopinatus*, *X. epipastus*, *X. subepipastus*, etc.), but share the common features like small basidiomata, squamulose pileus, epithelial pileipellis with globose to subglobose terminal elements, the presence of cheilocystidia, the absence of pleurocystidia, more or less yellow to brownish yellow basidiospores, and the absence of clamp connections. It should be noted that most species of the genus *Xanthagaricus* have ornamented basidiospores (figs 15c–h, 16 in Little Flower et al. 1997) but it was not mentioned in the genus circumscription by the authors (1997).

Macromorphologically, both new species are superficially close to each other, and could be confused in the field, although *X. flavosquamosus* has relatively larger squamules, no annulus and slightly lighter color that *X. necopinatus*. However, they can be separated microscopically. *Xanthagaricus flavosquamosus* has short and broadly clavate cheilocystidia, comparatively larger and wider basidiospores with a verrucose surface under SEM, while *X. necopinatus* has narrowly clavate cheilocystidia and shorter basidiospores with a rugulose-rough surface under SEM. Furthermore, these two species are in different clades in the phylogeny. *Xanthagaricus flavosquamosus* creates a new phyletic line with weak support, while *X. necopinatus* is a close relative to *X. pakistanicus* with strong BS support value (93% ML BS, Fig. 1). However, morphologically, *X. pakistanicus* has a light orange-yellow to moderate orange-yellow pileus, and globose to subglobose basidiospores measuring 7–7.5 × 6.5–7.0 µm (Hussain et al. 2017).

Some morphologically closely related species to be compared to *X. necopinatus* and *X. flavosquamosus* are *X. epipastus*, *X. ochraceoluteus* (D.A. Reid & Eicker) Hussain, *X. subepipastus* (Heinem. & Little Flower) Little Flower, Hosag. & T.K. Abraham, and *X. viridulus* (Heinem. & Little Flower) Little Flower, Hosag. & T.K. Abraham. The latter two species differ from *X. necopinatus* in having comparatively larger and wider basidiospores (Heinemann and Little Flower 1984, Daniëls et al. 2015). *Xanthagaricus epipastus* has an olive yellow to olivaceous squamulose pileus, and slightly wider basidiospores (3.7–4.7 × 2.8–3.4 µm) with lower Q value (avg. 1.36) (Heinemann and Little Flower 1984). *Xanthagaricus ochraceoluteus* differs from *X. necopinatus* in having olive-buff lamellae, no annulus, and variable cystidia. *Xanthagaricus flavosquamosus* has comparatively larger and wider basidiospores (5–5.5× 3–3.5 µm) than those of *X. epipastus* (see above), *X. subepipastus* (3.7–4.7 × 2.8–3.4) and *X. viridulus* (3.8–5.0 × 2.9–3.6 µm) (Heinemann and Little Flower 1984, Heinemann and Rammeloo 1986, Daniëls et al. 2015). Furthermore, *X. epipastus* has a pileus covered by olive yellow to olivaceous squamules (Heinemann and Little Flower 1984). Moreover, *X. subepipastus* and *X. viridulus*, originally described from the Kerala state of India, differ in having lageniform cheilocystidia (Heinemann and Little Flower 1984). *Xanthagaricus viridulus* also has an umbonate, brown, floccose-squamulose pileus, and lageniform to clavate cheilocystidia (Heinemann and Little Flower 1984). *Xanthagaricus ochraceoluteus* has olive-buff adnexed lamellae (Reid and Eicker 1998). Unfortunately, molecular data for the Indian collections and other comparable species are unavailable to include in this study.

*Xanthagaricus taiwanensis* (=*Hymenagaricus taiwanensis* Zhu L. Yang, Z.W. Ge & C.M. Chen), originally described from Taiwan, China is distinguished from *X. flavosquamosus* by having a yellow-brown pileus covered with fuscous brown-black squamules, a white membranous annulus, and comparatively wider basidiospores 5–5.5 × 3–4 µm (Ge et al. 2008). *Xanthagaricus caeruleus*, a recently described species from China, can also be distinguished from *X. flavosquamosus* by its grayish lilac to grayish violet squamules on pileus, ink-blue lamellae, and comparatively larger and smooth basidiospores 5–6 × 3–3.5 µm (Hosen et al. 2017). On the other hand, *X. necopinatus* is distinguished from all closely related species of this genus (see above), and the first contribution to the genus *Xanthagaricus* for Bangladesh.

It is interesting to note that *Xanthagaricus* appears to be a monophyletic genus and close sister to *Pseudolepiota* Z.W. Ge, a monotypic genus, recently described from China, with strong BS support value (85% ML BS). The latter genus is distinguished in having white color of the lamellae, hyaline basidiospores, and a subcutis pileipellis made up of slightly interwoven cylindrical hyphae (Ge and Yang 2017). However, the synapomorphic features of the two genera are the squamulose pileus, the absence of pleurocystidia, and the absence of clamp connections. Though a recent molecular study by Hosen et al. (2017) recovered *Xanthagaricus* as a close sister genus to *Hymenagaricus*, lacked significant BS support value while using ITS data. With the inclusion of eight species of *Xanthagaricus* including two new species based on ITS-28S phylogeny, the monophyly of the genus is resolved with the close evolutionary relationship to *Pseudolepiota*, and distinct from *Hymenagaricus* (Fig. 1).

The collection of *Heinemannomyces* made from south China matches well with the salient features (woolly-arachnoid veil on pileus, leaden gray lamellae, and a reddening context) of *H. splendidissimus* reported in the protologue by Watling (1998). However, the Chinese material slightly deviates from the original description in having comparatively larger basidiomata (up to 65 mm broad), and the absence of clamp connections. The authors were unable to include the type material of *Heinemannomyces* in the present study, but several sequences of *H. splendidissimus* from the type locality and its adjacent areas (Thailand) are available in the public accessible database (GenBank) to compare with the Chinese material. Sequences of *Heinemannomyces* from Thailand fall in the same clade with those from China, and are closely related to *Hymenagaricus* (Fig. 1). However, morphologically, *Heinemannomyces* differs from *Hymenagaricus* by having woolly-arachnoid veil remnants on the pileus surface, becoming brown, a reddening context when cut or injured, leaden gray lamellae, and a pileipellis composed of cylindrical hyphae (Watling 1998). It should be noted that three species of *Hymenagaricus* (GenBank voucher numbers. C.M. Chen 3636, T. Laessoe 6025 and ecv3807) used in this study (*Xanthagaricus* clade, Fig. 1) were also grouped together with strong BS support value in the molecular study of Vellinga et al. (2011) based on either ITS or multigene phylogeny, and *Heinemannomyces* was separated from them, and formed an independent lineage (Figs 2 and 3 in Vellinga et al. 2011). However, no additional species of *Hymenagaricus* from the *Hymenagaricus*-*Heinemannomyces* clade (Fig. 1) was included in the study of Vellinga et al. (2011). In the present analysis, more species of *Hymenagaricus* including some other close relative taxa of Agaricaceae were included, and *Heinemannomyces* showed a close affinity to *Hymenagaricus* (Fig. 1). One possible classification would be to collapse *Heinemannomyces* into a single genus *Hymenagaricus* or separate it into a subgenus/section. However, there are some remarkable morphological characteristics for *Heinemannomyces* and *Hymenagaricus*, which supports separating them into different genera. Further exploration of *Hymenagaricus*/*Heinemannomyces* species diversity and historical biogeography from Asia/South Africa, which seems to be species rich in these genera, could help to confirm or refute the hypothesis of monophyly/paraphyly of *Hymenagaricus*.

### Key to the taxa of *Xanthagaricus* known from Bangladesh and China

**Table:** 

1	Basidiomata small (8–15 mm broad)	**2**
–	Basidiomata small (15–35 mm broad), with yellow brown pileus, covered with fuscous black squamules, lamellae pink becoming grayish pink, basidiospores 5–5.5 × 3–4 µm, smooth, pileipellis epithelial with encrusted wall, found in Taiwan, China	***X. taiwanensis***
2	Basidiomata small (10–15 mm broad), with dull lilac to grayish lilac or grayish violet squamules, lamellae white becoming light blue to blackish blue, basidiospores 5–6 × 3–3.5 µm, smooth surface under SEM, pileipellis epithelial without encrusted wall but pigmented, found in China	***X. caeruleus***
–	Basidiomata small (8–15 mm broad), with yellow to yellowish brown, lamellae yellowish white to light pinkish white, pileipellis epithelial with encrusted wall, basidiospores 4–5.5 × 2.7–3.5 µm, ornamented under SEM	**3**
3	Basidiomata 8–13 mm broad, basidiospores 5–5.5 × 3–3.5 µm, ornamented with verrucose surface under SEM, fugacious annulus absent, found in China	***X. flavosquamosus***
–	Basidiomata 10–15 mm broad, basidiospores 4–5 × 2.7–3.2 µm, ornamented with rugulose-rough surface under SEM, fugacious annulus present, found in Bangladesh	***X. necopinatus***

## Supplementary Material

XML Treatment for Xanthagaricus flavosquamosus

XML Treatment for Xanthagaricus necopinatus

XML Treatment for Heinemannomyces splendidissimus
